# A twist for survival and cancer progression

**DOI:** 10.1038/sj.bjc.6602876

**Published:** 2005-11-22

**Authors:** A Puisieux, S Valsesia-Wittmann, S Ansieau

**Affiliations:** 1 INSERM U590 Centre Léon Bérard, Université Claude Bernard Lyon 1, 69373 Lyon Cedex 08, France

**Keywords:** twist-1, neuroblastoma, breast cancer, apoptosis, metastasis

## Abstract

A major obstacle to the expansion of abnormal cells with significant proliferative potential is the induction of programmed cell death. Consequently, oncogene-driven hyperproliferation must be associated with apoptosis inhibition to allow malignant outgrowth. The oncogenic cooperation of N-Myc and Twist-1 in the development of neuroblastoma, the most common and deadly solid tumour of childhood, perfectly illustrates such a process. N-Myc promotes cell proliferation, whereas Twist-1 counteracts its pro-apoptotic properties by knocking-down the ARF/p53 pathway. On the basis of numerous recent studies reporting its overexpression in a variety of human cancers, we discuss in this review the role of Twist-1 as a potent inhibitor of the cell safety programs engaged in response to an abnormal mitogenic activity.

The Twist-1 protein (also called Twist) is a highly conserved transcription factor that belongs to the family of basic helix–loop–helix (bHLH) proteins (Thisse *et al*, 1987). bHLH proteins are structurally and functionally characterised by a conserved domain containing a stretch of basic amino acids adjacent to two amphipathic *α*-helices separated by an interhelical loop. The *α*-helices mediate dimerisation with a second bHLH factor, leading to the formation of a bipartite DNA-binding domain that specifically binds to hexanucleotide sequences (CANNTG), referred to as E-boxes. Functional E-boxes are found in the regulatory elements of many lineage-specific genes. Consistently, bHLH are transcription factors acting in various differentiation processes, either as positive or negative regulators, and play key roles in different developmental events like neurogenesis and myogenesis (for review see Jan and Jan, 1993). *Drosophila twist* is a zygotic developmental gene involved in early mesoderm patterning (Thisse *et al*, 1987). It is regulated by a nuclear gradient of the protein Dorsal, a Rel-containing sequence-specific transcription factor (Ip *et al*, 1992), leading to *twist* expression in the ventral mesoderm. The encoded Twist protein forms a steep gradient across the presumptive mesoderm–neuroectoderm border in the early embryo. *Drosophila* twist mutants fail to gastrulate indicating that it is not only necessary for mesoderm differentiation but also for the morphogenetic movement during gastrulation (Leptin, 1991). Two *Twist* genes exist in vertebrates, *Twist-1* (*Twist*) and *Twist-2* (formerly known as *Dermo-1*) (Li *et al*, 1995). The two Twist proteins display more than 90% identity in the bHLH and carboxy-terminal domains (Figure 1). The C-terminal part of the proteins comprises a ‘Twist box’ which is involved in the antiosteogenic function of Twist-1 and Twist-2 (Bialek *et al*, 2004). The N-termini of Twist-1 and Twist-2 are more divergent, and Twist-2 lacks a glycine-rich region that is present in Twist-1 (Li *et al* 1995). Twist-1 and Twist-2 have been implicated in the differentiation of multiple cell lineages including muscle, cartilage and osteogenic cells. In mice, *Twist-1* was shown to be required for proper development of the head mesenchyme, somites and limb buds. Mice lacking *Twist-1* die at E10.5, whereas mice homozygous for a *Twist-2* null allele show elevated expression of pro-inflammatory cytokines causing perinatal death (Sosic *et al*, 2003). Interestingly, mice heterozygous for *Twist-1* or *Twist-2* mutant alleles are viable, and double heterozygous mutants phenocopy the severe cachexia seen in *twist-2* null mice, suggesting that *Twist-1* and *Twist-2 may* act redundantly to control an essential post-natal process (Sosic *et al*, 2003). Heterozygosity for *Twist-1*-null mutations results in a moderate phenotype including minor skull and limb anomalies. Consistently, germ-line mutations of the coding sequence of the human *Twist-1* gene, leading to haploinsufficiency, have been identified in the Saethre–Chotzen syndrome (SCS) (acrocephalosyndactyly type III, ACS III; MIM No.101400). SCS is a hereditary disorder transmitted as an autosomal dominant trait and characterised by abnormalities of the limbs, asymmetric head and face, and premature fusion of cranial sutures (Pantke *et al*, 1975).

Besides this important role in differentiation, various observations strongly support a regulatory effect of Twist proteins on programmed cell death. Notably, *Twist-1* haploinsufficiency in SCS induces calvarial osteoblast apoptosis and *Twist-1* homozygous inactivation in animal models induces massive apoptosis during mammalian development (Chen and Behringer, 1995). *Twist-2*^−/−^ cells are also dramatically sensitised to cytokine-induced apoptosis (Sosic *et al*, 2003). *In vitro* experiments on mouse embryo fibroblasts further showed that Twist-1 interferes with the ARF-p53 regulation pathway, thus blocking c-myc-induced apoptotic response (Maestro *et al*, 1999). Moreover, DNazyme-mediated cleavage of mouse *Twist-1* transcripts in mouse mesenchymal C3H10T1/2 cells increases apoptosis (Hjiantoniou *et al*, 2003).

## TWIST, APOPTOTIC FAILSAFE PROGRAM AND CANCER: THE MODEL OF NEUROBLASTOMA

Neuroblastoma (NB) stands out among paediatric solid tumours because of its relative frequency, intriguing natural history, prognostic biologic features, and therapeutic challenges (Brodeur, 2003). Neuroblastoma is considered as an embryonal malignancy of the postganglionic sympathetic nervous system, generally arising in the adrenal gland. It is the most common extracranial paediatric solid tumour, accounting for 7–10% of all childhood cancers, and the most common neoplasm in infancy. NB is characterised by a highly heterogeneous clinical behaviour, ranging from spontaneous regression without treatment to aggressive therapy-resistant disease. At present, patients are classified into risk categories on the basis of a combination of clinical and biological markers. These include age at diagnosis, stage of disease, histopathology findings, and amplification of the proto-oncogene *N-Myc*. *N-Myc* amplification, observed in about 25% of primary tumours, is strongly associated with rapid tumour progression and a poor outcome, independently from the stage of the tumour or the age of the patient, and has thus become an important factor in clinical decision-making and therapy (Brodeur, 2003). Myc oncoproteins are known examples of cellular oncoproteins that act both as growth promoting factors and apoptosis promoters (Evan *et al*, 1992). Apoptosis is mainly triggered by the ARF-p53 pathway (Zindy *et al*, 1998). Interestingly, an *in vivo* analysis of p53-dependent functions in Myc-induced lymphomas arising in E*μ*-*myc* transgenic mice has indicated that apoptosis is the only p53 effector program that is selected against during tumour formation (Schmitt *et al*, 2002), suggesting that cell cycle checkpoint defects and genomic instability are by-products of *p53* loss. Apoptosis is therefore a crucial failsafe program to counteract oncogenic Myc activation, and tumour development requires both hyperproliferation and prosurvival signalling. Intriguingly, in contrast with most human cancer types exhibiting an oncogenic Myc activation, primary NBs rarely display *p53* mutations, and deletions or loss of heterozygosity (LOH) at 17p (where *p53* maps) are uncommon (Vogan *et al*, 1993). This suggests that other defects in p53-dependent pathways may substitute for intragenic *p53* mutations. Although amplification of the p53-inhibitory *MDM2* locus has been identified in rare cases of NBs and abnormal cytoplasmic p53 localisation has been observed by immunohistochemistry in undifferentiated NBs (for review see Borriello *et al*, 2002), the mechanism of inhibition of p53-dependent apoptosis remains largely unknown. Interestingly, a deregulation of important regulators of apoptosis has been reported in NBs, including overexpression of survivin, a member of the IAP family, and down-expression of caspase 8. However, no obvious correlation with *N-Myc* amplification has been observed (Borriello *et al*, 2002).

**Figure 2:**
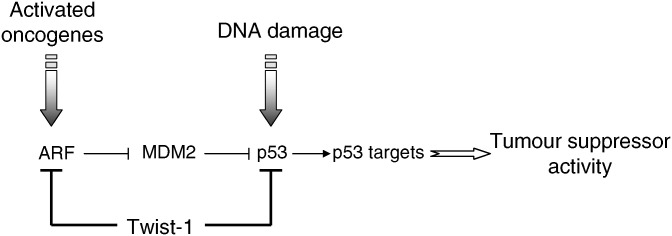
Twist-1 as a potent inhibitor of p53 tumour suppressor activity. Twist inhibits p53-mediated response to cellular stress (activated oncogenes or DNA damage) by modulating both *ARF* expression and p53 post-translational modifications such as phosphorylation and acetylation.

*N-Myc*-amplified NBs are thus remarkable examples of tumours displaying a high mitogenic activity, in the presence of a wild-type p53. With the aim to identify oncogenes or tumour suppressor genes whose alterations are able to cooperate with N-Myc in NB development, we performed a genome-wide microarray analysis of human NBs (Valsesia-Wittmann *et al*, 2004). This study showed that *N-Myc* amplification was constantly associated to the overexpression of *Twist-1*. On the basis of previous observations suggesting an anti-apoptotic activity of Twist-1, we hypothesised that Twist-1 was able to interfere with the cellular mechanism that triggers apoptosis in NB cells overexpressing *N-Myc*. This hypothesis was first verified by inhibiting endogenous *Twist-1* expression in NB cell lines endogenously overexpressing *N-Myc* and *Twist-1*. Such inactivation by RNA interference was sufficient to promote massive apoptosis. In line with this observation, forced expression of *Twist-1* in non *N-Myc* amplified NB cells was able to inhibit p53 response to genotoxic stress. We next demonstrated that N-Myc and Twist-1 co-expression transforms early-passage mouse embryo fibroblasts (MEFs), whereas each oncogene separately fails to do so. The oncogenic cooperation between N-Myc and Twist-1 was finally confirmed *in vivo*, the co-expression of the two oncogenes increasing tumourigenicity of human NB cells after injection in *nude* mice. Overall, this study demonstrated the oncogenic cooperation of two major regulators of embryogenesis, N-Myc and Twist-1, in the development of NB, supporting the current hypothesis of an embryonic origin of this tumour. N-Myc induces cell proliferation, whereas Twist-1 inhibits the ARF-p53 dependent pathway and prevents the apoptotic response normally triggered by Myc overexpression. By identifying *Twist-1* overexpression as a cause of p53 inactivation, we not only provide a mechanistic explanation for the rarity of *p53* mutations in NBs, but also show that the collaboration between N-Myc and Twist-1 is based on escape from natural safeguard response. Nevertheless, the mechanisms by which Twist-1 compromises p53-response remain elusive. Several observations suggest that Twist-1 may act at different steps of p53-regulation including transcriptional control and post-translational modifications (Figure 2). Recently, the group of Raman demonstrated that Twist-1 was able to physically interact with HOXA5, a potent transactivator of the *p53* promoter, and to modulate its activity (Stasinopoulos *et al*, 2005). Twist-1 was also found to affect p53 stabilisation by reducing the expression of the *ARF* tumour suppressor, an upstream regulator of p53 (Maestro *et al*, 1999), and to prevent post-translational modifications, such as serine 20 phosphorylation, essential for p53 activation in response to DNA damage (Stasinopoulos *et al*, 2005). Finally, Twist-1 could also inhibit p53-mediated gene transcription through its demonstrated ability to inhibit p300-mediated acetylation, another post-translational process involved in the control of p53 activity (Hamamori *et al*, 1999).

Recent data demonstrated that the anti-apoptotic role of Twist-1 is not restricted to NB cells. Indeed, *Twist-1* overexpression was reported in a variety of solid cancers including breast, prostate and gastric carcinomas, melanomas, osteosarcomas, rhabdomyosarcomas, as well as in Sezary syndrome, a malignancy of CD4+ memory skin-homing T cells (Maestro *et al*, 1999; Rosivatz *et al*, 2002; Hoek *et al*, 2004; van Doorn *et al*, 2004; Watanabe *et al*, 2004; Entz-Werle *et al*, 2005; Kwok *et al*, 2005; Martin *et al*, 2005). Of note, *Twist-2* has been shown recently to be deregulated in chronic lymphocytic leukaemia (Raval *et al*, 2005). Experimental downregulation of *Twist-1* through small interfering RNA promotes apoptosis in human breast cancer and melanoma cell lines (unpublished data). Interestingly, the downregulation of *Twist-1* increases cell death sensitivity of PC3 and DU145 prostate cancer cells, that both lack functional p53, suggesting that Twist-1 might regulate apoptosis through both p53-dependent and p53-independent pathways (Kwok *et al*, 2005). As mentioned previously, *Twist-2*^−/−^ cells both upregulate TNF*α* expression and are sensitised to TNF*α*-induced apoptosis (Sosic *et al*, 2003). This phenotype is recapitulated in *Twist-1* and *Twist-2* compound heterozygotes reflecting redundancy and dosage dependence of these genes in inhibition of cytokine expression (Sosic *et al*, 2003). In addition to inactivating p53, Twist proteins may thus also act as NF-*κ*B-controlled anti-apoptotic factors.

Considering that *N-Myc* amplified NBs are highly metastatic tumours and that *Twist-1* overexpression in melanomas is associated to an aggressive phenotype (Hoek *et al*, 2004), we hypothesise that the anti-apoptotic activity of Twist-1 is likely to play a significant role in the metastatic process. Our hypothesis is strengthened by previous reports demonstrating that abrogation of p53-mediated apoptosis facilitates experimental metastasis by promoting the survival of tumour cells in circulation (Nikiforov *et al*, 1996). The overexpression of *Twist-1* may thus be a selective advantage for a primary tumour cell to survive in settings of limited survival signals when contact with the original tumour is lost. Indeed, besides the necessity to avoid effective immune clearance and to survive in multiple contexts (local stroma, blood or lymph, secondary site of expansion), malignant cells have to override a significant number of barriers to metastasise. Hence, the metastatic process requires a complex set of cellular functions, including changes in adhesion, initiation of motility, extracellular matrix proteolysis, intravasation, extravasation into the surrounding tissue, molecular interactions of cancer cells with the environment of the secondary site, initiation of angiogenesis for ensuring independent nutrient supply and maintenance of growth leading to the metastatic colonisation.

## TWIST, EMT AND METASTASIS: THE MODEL OF BREAST CARCINOMA

Although still a subject of controversy (Thompson and Newgreen, 2005; Tarin, 2005), the epithelial–mesenchymal transition (EMT) is proposed as an important step of carcinoma progression. EMT is a variant of transdifferentiation and a mechanism for dispersing cell lineages in vertebrate embryos. It is seen early in the course of development during gastrulation, and plays a major role during the formation of the heart, the musculoskeletal system, most craniofacial structures and the peripheral nervous system. Twist participates in EMTs during mesoderm differentiation in *Drosophila* and during neural crest migration in vertebrates. Hallmarks of developmental EMT include derangements of apicobasal polarity, lack of basal laminal integrity, and cell shape plasticity, allowing an increased migration capability. These processes are associated to a switch from E-cadherin (epithelial cadherin) to N-cadherin (neural cadherin) expression. E-cadherin is a homotypic cell–cell adhesion molecule which is required for the formation of epithelia in the embryo. It is then expressed on most epithelial cells, where it forms the adherens junction and facilitates the formation of the entire epithelial junctional complex. N-cadherin typically forms homotypic homophilic interactions, but also heterotytpic and heterophilic interactions have been described. In the early embryo, N-cadherin is found in the mesoderm, whereas in the late embryo it is present in neural tissue, lens and several other epithelial tissues (Derycke and Bracke, 2004). In the adult, N-cadherin expression is mainly restricted to neural tissue, retina, endothelial cells, fibroblasts and myocytes. Because strong migratory and invasive abilities are also characteristic of metastatic cells, it has been suggested that epithelial cell-derived tumours acquire a mesenchymal character during tumour progression. Indeed, several genes implicated in EMT during embryogenesis are deregulated during tumorigenesis, including the zinc-finger proteins Snail, SIP-1 and Slug that act as E-cadherin repressors (Huber *et al*, 2005, for review). Loss of E-cadherin-mediated cell–cell adhesion is a frequent event during tumour cell invasion and metastasis formation (Heimann *et al*, 2000). Consistent with this finding, noninvasive transformed cells *in vitro* become invasive upon abolishing E-cadherin function or expression (Vleminckx *et al*, 1991). Conversely, transfection of highly invasive epithelial tumor cell lines by E-cadherin cDNA totally abrogates their invasiveness potential. Several mechanisms are found to underlie the loss of E-cadherin function during tumorigenesis: these include mutational events, mostly in breast lobular carcinomas, or epigenetic mechanisms such as promoter hypermethylation or transcriptional repression of the gene by repressor factors. Conversely, gain of expression of N-cadherin is frequently observed in carcinomas and its forced expression promotes motility and metastasis of prostate and breast cancer cells. Interestingly, *Twist* has been shown to be essential for the initiation of *Drosophila* N-cadherin expression during gastrulation (Oda *et al*, 1998), and *Twist-1* overexpression is correlated to abnormal expression of N-cadherin mRNA in human diffuse-type gastric cancer (Rosivatz *et al*, 2002). As previously mentioned, *Twist-1* is upregulated in several types of epithelial cancers including breast, prostate and gastric carcinomas (Rosivatz *et al*, 2002; Watanabe *et al*, 2004; Kwok *et al*, 2005; Martin *et al*, 2005). Although the mechanisms underlying this overexpression remain unknown, *Twist-1* expression has been shown to be induced in response to Wnt-1 in mouse mammary epithelial cells, suggesting that it might be regulated by Wnt signalling (Howe *et al*, 2003). Recently, by using a mouse mammary tumour model, in which a set of otherwise isogenic tumour cell populations is able to complete distinct steps of metastasis when implanted into the mammary glands of BALB/c mice, the group of R. Weinberg showed that the murine homologue of Twist-1 was required for intravasation and metastasis of breast cancer cells in the lung (Yang *et al*, 2004). Indeed, inhibition of *Twist-1* expression by RNAi in metastatic cancer cells significantly decreases the efficiency of lung metastasis from the mammary gland as well as the number of tumour cells that could be recovered from the circulation. The same authors also demonstrated that ectopic expression of *Twist-1* leads to molecular and morphological features associated with an EMT, including induction of cell motility, loss of E-cadherin-mediated cell–cell adhesion and activation of mesenchymal markers. Similar observations were recently reported in androgen-independent prostate cancer cells (Kwok *et al*, 2005). The link between Twist-1 and E-Cadherin was strengthened by the demonstration that Twist-1, directly or indirectly, causes the transcriptional repression of E-cadherin through the E-box elements present in its promoter region (Yang *et al*, 2004). Nevertheless, the reported observation that *Twist-1-*overexpression is more frequent in breast lobular carcinomas than in breast ductal carcinomas (Yang *et al*, 2004) is intriguing as more than 50% of lobular carcinomas have E-cadherin mutations. The fact that forced expression of E-cadherin is not sufficient to reverse the process in *Twist-1*-expressing cells (Yang *et al*, 2004) further supports the hypothesis that Twist-1 might modulate several important processes during cancer development, including inhibition of cell safeguard programs, alteration of cell–cell adhesion (through control of E- and N-cadherin expression) and deregulation of differentiation, leading to both cell survival and invasion capability.

## CONCLUDING REMARKS

Over the last year, numerous studies have reported the upregulation of *Twist-1* in a variety of human cancers. Functional studies have indicated that Twist-1 may play a major role in tumour promotion and progression, by inhibiting differentiation, interfering with the p53 tumour suppressor pathway and favouring cell survival, and/or inducing an epithelial–mesenchymal-like transition. Although further studies required to determine the exact nature of Twist-1 involvement in a defined cancer type, this novel identification of a crucial regulator of embryogenesis as an oncogene supports the view of tumours as corrupt forms of normal development.

At last, the observation of Twist-1 activation in human cancers might have important clinical implications, both as a prognostic and/or predictive factor and as a novel therapeutic target. In support of the first hypothesis, *Twist-1* overexpression was shown, recently, to be a factor of poor prognosis in melanomas (Hoek *et al*, 2004), and *Twist-1* forced expression triggers the resistance of human cancer cells to microtubule agents such as taxol and vincristine (Wang *et al*, 2004; Kwok *et al*, 2005). Furthermore, as discussed previously, Twist-1 probably inhibits both p53-dependent and p53-independent apoptosis. Consequently, the experimental inactivation of *Twist-1* by RNA interference leads to cell death in a variety of cancer cell lines, including NB, melanoma and breast cancer lines. Downregulation of *Twist-1* by antisense technology in mouse fibroblasts also increases the sensitivity to etoposide, a DNA-damaging anticancer drug (Dupont *et al*, 2001). Overall, these observations suggest that Twist-1 might be an interesting therapeutic target for increasing cell sensitivity to drug-induced apoptosis.

## Figures and Tables

**Figure 1:**
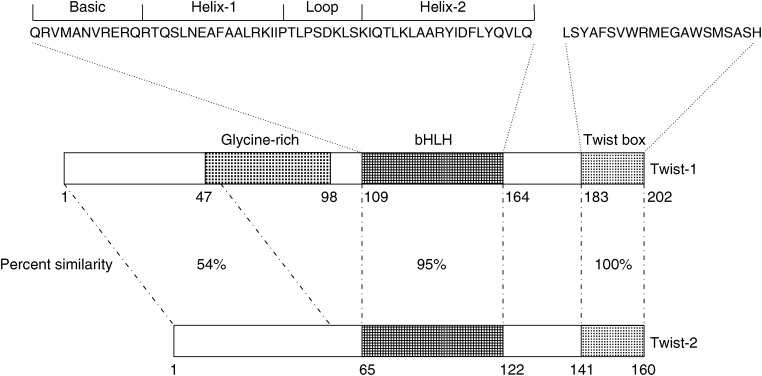
Functional domains of human Twist-1 and Twist-2 proteins. The glycine-rich region is specific to Twist-1. The Runx2-binding ‘Twist box’ has been implicated in the antiosteogenic function of Twist-1 and Twist-2 (Bialek *et al*, 2004).

## References

[bib1] Bialek P, Kern B, Yang X, Schrock M, Sosic D, Hong N, Wu H, Yu K, Ornitz DM, Olson EN, Justice MJ, Karsenty G (2004) A twist code determines the onset of osteoblast differentiation. Dev Cell 6: 423–435 15030764 10.1016/s1534-5807(04)00058-9

[bib2] Borriello A, Roberto R, Della Ragione F, Iolascon A (2002) Proliferate and survive: cell division cycle and apoptosis in human neuroblastoma. Haematologica 87: 196–214 11836171

[bib3] Brodeur GM (2003) Neuroblastoma: biological insights into a clinical enigma. Nat Rev Cancer 3: 203–216 12612655 10.1038/nrc1014

[bib4] Chen ZF, Behringer RR (1995) *Twist* is required in head mesenchyme for cranial neural tube morphogenesis. Genes Dev 9: 686–699 7729687 10.1101/gad.9.6.686

[bib5] Derycke LDM, Bracke ME (2004) N-cadherin in the spotlight of cell–cell adhesion, differentiation, embryogenesis, invasion and signalling. Int J Dev Biol 48: 463–476 15349821 10.1387/ijdb.041793ld

[bib6] Dupont J, Fernandez AM, Glackin CA, Helman L, LeRoith D (2001) Insulin-like growth factor 1 (IGF-1)-induced twist expression is involved in the anti-apoptotic effects of the IGF-1 receptor. J Biol Chem 276: 26699–26707 11323435 10.1074/jbc.M102664200

[bib7] Entz-Werle N, Stoetzel C, Berard-Marec P, Kalifa C, Brugiere L, Pacquement H, Schmitt C, Tabone MD, Gentet JC, Quillet R, Oudet P, Lutz P, Babin-Boilletot A, Gaub MP, Perrin-Schmitt F (2005) Frequent genomic abnormalities at TWIST in human pediatric osteosarcomas. Int J Cancer 117: 349–355 15900593 10.1002/ijc.21068

[bib8] Evan GI, Wyllie AH, Gilbert CS, Littlewood TD, Land H, Brooks M, Waters CM, Penn LZ, Hancock DC (1992) Induction of apoptosis in fibroblasts by c-myc protein. Cell 69: 119–128 1555236 10.1016/0092-8674(92)90123-t

[bib9] Hamamori Y, Sartorelli V, Ogrysko V, Puri PL, Wu H-Y, Wang JYJ, Nakatani Y, Kedes L (1999) Regulation of HAT p300 and pCAF by the bHLH Twist and adenoviral oncoprotein E1A. Cell 96: 405–413 10025406 10.1016/s0092-8674(00)80553-x

[bib10] Heimann R, Lan F, McBride R, Hellman S (2000) Separating favourable from unfavourable prognostic markers in breast cancer: the role of E-cadherin. Cancer Res 60: 298–304 10667580

[bib11] Hjiantoniou E, Iseki S, Uney JB, Phylactou LA (2003) DNazyme-mediated cleavage of Twist transcripts and increase in cellular apoptosis. Biochem Biophys Res Commun 300: 178–181 12480539 10.1016/s0006-291x(02)02804-8

[bib12] Hoek K, Rimm DL, Williams KR, Zhao H, Ariyan S, Lin A, Kluger HM, Berger AJ, Cheng E, Trombetta ES, Wu T, Niinobe M, Yoshikawa K, Hannigan GE, Halaban R (2004) Expression profiling reveals novel pathways in the transformation of melanocytes to melanomas. Cancer Res 64: 5270–5282 15289333 10.1158/0008-5472.CAN-04-0731

[bib13] Howe LR, Watanabe O, Leonard J, Brown AMC (2003) Twist is up-regulated in response to Wnt1 and inhibits mouse mammary cell differentiation. Cancer Res 63: 1906–1913 12702582

[bib14] Huber MA, Kraut N, Beug H (2005) Molecular requirements for epithelial–mesenchymal transition during tumor progression. Curr Opin Cell Biol 17: 548–558 16098727 10.1016/j.ceb.2005.08.001

[bib15] Ip YT, Park RE, Kosman D, Bier E, Levine M (1992) The dorsal gradient morphogen regulates stripes of rhomboid expression in the presumptive neuroectoderm of the *Drosophila* embryo. Genes Dev 6: 1728–1739 1325394 10.1101/gad.6.9.1728

[bib16] Jan YN, Jan LY (1993) Functional gene cassettes in development. Proc Natl Acad Sci USA 90: 8305–8307 8378299 10.1073/pnas.90.18.8305 PMC47343

[bib17] Kwok WK, Ling MT, Lee TW, Lau TC, Zhou C, Zhang X, Chua CW, Chan KW, Chan FL, Glackin C, Wong YC, Wang X (2005) Up-regulation of TWIST in prostate cancer and its implication as a therapeutic target. Cancer Res 65: 5153–5162 15958559 10.1158/0008-5472.CAN-04-3785

[bib18] Leptin M (1991) twist and snail as positive and negative regulators during Drosophila mesoderm development. Genes Dev 5: 1568–1576 1884999 10.1101/gad.5.9.1568

[bib19] Li L, Cserjesi P, Olson EN (1995) Dermo-1: a novel twist-related bHLH protein expressed in the developing dermis. Dev Biol 172: 280–292 7589808 10.1006/dbio.1995.0023

[bib20] Maestro R, Dei Tos AP, Hamamori Y, Krasnokutsky S, Sartorelli V, Kedes L, Doglioni C, Beach DH, Hannon GJ (1999) Twist is a potential oncogene that inhibits apoptosis. Genes Dev 13: 2207–2217 10485844 10.1101/gad.13.17.2207 PMC317004

[bib21] Martin TA, Goyal A, Watkins G, Jiang WG (2005) Expression of the transcription factors snail, slug, and twist and their clinical significance in human breast cancer. Ann Surg Oncol 12: 488–496 15864483 10.1245/ASO.2005.04.010

[bib22] Nikiforov MA, Hagen K, Ossovskaya VS, Connor TM, Lowe SW, Deichman GI, Gudkov AV (1996) p53 modulation of anchorage independent growth and experimental metastasis. Oncogene 13: 1709–1719 8895517

[bib23] Oda H, Tsukita S, Takeichi M (1998) Dynamic behavior of the cadherin-based cell-cell adhesion system during Drosophila gastrulation. Dev Biol 203: 435–450 9808792 10.1006/dbio.1998.9047

[bib24] Pantke OA, Cohen Jr MM, Witkop Jr CJ, Feingold M, Schaumann B, Pantke HC, Gorlin RJ (1975) The Saethre–Chotzen syndrome. Birth Defects Orig Arti Ser 11: 190–225 1227525

[bib25] Raval A, Lucas DM, Matkovic JJ, Bennett KL, Liyanarachchi S, Young DC, Rassenti L, Kipps TJ, Grever MR, Byrd JC, Plass C (2005) TWIST2 demonstrates differential methylation in immunoglobulin variable heavy chain mutated and unmutated chronic lymphocytic leukemia. J Clin Oncol 23: 3877–3885 15809452 10.1200/JCO.2005.02.196

[bib26] Rosivatz E, Becker I, Specht K, Fricke E, Luber B, Busch R, Hofler H, Becker KF (2002) Differential expression of the epithelial–mesenchymal transition regulators snail, SIP1, and twist in gastric cancer. Am J Pathol 161: 1881–1891 12414534 10.1016/S0002-9440(10)64464-1 PMC1850763

[bib27] Schmitt CA, Fridman JS, Yang M, Baranov E, Hoffman RM, Lowe SW (2002) Dissecting p53 tumor suppressor functions *in vivo*. Cancer Cell 1: 289–298 12086865 10.1016/s1535-6108(02)00047-8

[bib28] Sosic D, Richardson JA, Yu K, Ornitz DM, Olson EN (2003) Twist regulates cytokine gene expression through a negative feedback loop that represses NF-B activity. Cell 112: 169–180 12553906 10.1016/s0092-8674(03)00002-3

[bib29] Stasinopoulos IA, Mironchik Y, Raman A, Wildes F, Winnard Jr P, Raman V (2005) HOXA5-twist interaction alters p53 homeostasis in breast cancer cells. J Biol Chem 280: 2294–2299 15545268 10.1074/jbc.M411018200

[bib30] Tarin D (2005) The fallacy of epithelial mesenchymal transition in neoplasia. Cancer Res 65: 5996–6000 16024596 10.1158/0008-5472.CAN-05-0699

[bib31] Thisse B, Stoetzel C, El Messal M, Perrin-Schmitt F (1987) The twist gene: isolation of a *Drosophila* zygotic gene necessary for the establishment of dorso-ventral pattern. Nucleic Acids Res 15: 3439–3453 3106932 10.1093/nar/15.8.3439 PMC340740

[bib32] Thompson EW, Newgreen DF (2005) Carcinoma invasion and metastasis: a role for epithelial-mesenchymal transition? Cancer Res 65: 5991–5995 16024595 10.1158/0008-5472.CAN-05-0616

[bib33] Valsesia-Wittmann S, Magdeleine M, Dupasquier S, Garin E, Jallas AC, Combaret V, Krause A, Leissner P, Puisieux A (2004) Oncogenic cooperation between H-Twist and N-Myc overrides failsafe programs in cancer cells. Cancer Cell 6: 625–630 15607966 10.1016/j.ccr.2004.09.033

[bib34] van Doorn R, Dijkman R, Vermeer MH, Out-Luiting JJ, van der Raaij-Helmer EM, Willemze R, Tensen CP (2004) Aberrant expression of the tyrosine kinase receptor EphA4 and the transcription factor twist in Sezary syndrome identified by gene expression analysis. Cancer Res 64: 5578–5586 15313894 10.1158/0008-5472.CAN-04-1253

[bib35] Vleminckx K, Vakaet L, Mareel M, Fiers W, Van Roy F (1991) Genetic manipulation of E-cadherin expression by epithelial tumor cells reveals an invasion suppressor role. Cell 66: 107–119 2070412 10.1016/0092-8674(91)90143-m

[bib36] Vogan K, Bernstein M, Leclerc JM, Brisson L, Brossard J, Brodeur GM, Pelletier J, Gros P (1993) Absence of p53 gene mutations in primary neuroblastomas. Cancer Res 53: 5269–5273 8221661

[bib37] Wang X, Ling MT, Guan XY, Tsao SW, Cheung HW, Lee DT, Wong YC (2004) Identification of a novel function of TWIST, a bHLH protein, in the development of acquired taxol resistance in human cancer cells. Oncogene 23: 474–482 14724576 10.1038/sj.onc.1207128

[bib38] Watanabe O, Imamura H, Shimizu T, Kinoshita J, Okabe T, Hirano A, Yoshimatsu K, Konno S, Aiba M, Ogawa K (2004) Expression of twist and wnt in human breast cancer. Anticancer Res 24: 3851–3856 15736421

[bib39] Yang J, Mani SA, Donaher JL, Ramaswamy S, Itzykson RA, Come C, Savagner P, Gitelman I, Richardson A, Weinberg RA (2004) Twist, a master regulator of morphogenesis, plays an essential role in tumor metastasis. Cell 117: 927–939 15210113 10.1016/j.cell.2004.06.006

[bib40] Zindy F, Eischen CM, Randle DH, Kamijo T, Cleveland JL, Sherr CJ, Roussel MF (1998) Myc signalling via the ARF tumor suppressor regulates p53-dependent apoptosis and immortalization. Genes Dev 12: 2424–2433 9694806 10.1101/gad.12.15.2424 PMC317045

